# High stakes: how legal cannabis pricing impacts the illicit market

**DOI:** 10.1186/s42238-026-00455-3

**Published:** 2026-06-06

**Authors:** Rebecca Hebert, Kevin F. Boehnke, Spruha Joshi

**Affiliations:** 1 Department of Epidemiology, University of Michigan, Ann Arbor, MI USA; 2 Michigan Psychedelic Center, University of Michigan, Ann Arbor, MI USA; 3 Chronic Pain & Fatigue Research Center, University of Michigan, Ann Arbor, MI USA

**Keywords:** Cannabis prices, Illicit drug markets, Recreational cannabis legalization, Cannabis policy

## Abstract

**Background:**

Although nearly 80% of Americans reside in a county with a legal, regulated cannabis dispensary, the majority of cannabis consumed in the U.S. is still obtained from unregulated, illicit sources. Illicit cannabis carries greater health risks and is generally less preferred than regulated cannabis, yet its lower price makes it attractive to many customers. However, comparative research on price differentials between the legal and illicit markets across states is scarce. Establishing cannabis price benchmarks is essential to ensure that legal markets are competitively priced and can effectively displace the illicit trade.

**Methods:**

We used crowdsourced data from *PriceofWeed.com* to estimate the cost of illicit cannabis in 2018–2024 and data from state government websites to estimate the cost of legal cannabis in Colorado, Oregon, California, Massachusetts, Michigan, Illinois, Maine, and Connecticut. We compared illicit prices by recreational dispensary status in all 50 states and D.C. and examined legal vs. illicit prices within the eight aforementioned recreational states.

**Results:**

The median price per gram of illicit cannabis was significantly lower in recreational states, compared to non-recreational states ($7.10 vs. $8.90, *p* < 0.001). While illicit and legal prices were not significantly different over the entire study period, there were significant differences within years and within states. In Colorado, California, and Michigan, legal cannabis was significantly less expensive than illicit cannabis, while in Massachusetts, Illinois, Maine, and Connecticut, illicit cannabis was significantly less expensive than legal cannabis.

**Conclusions:**

We saw lower illicit market prices in states with recreational dispensaries. Our results suggest that the price ranking of legal versus illicit cannabis within a given state differs depending on the state’s recreational market maturity, with states with older markets – all of which also had more flexible licensing policies – having a legal cannabis price advantage.

**Supplementary Information:**

The online version contains supplementary material available at 10.1186/s42238-026-00455-3.

## Introduction

As of August 2025, 24 states and Washington D.C. have enacted recreational cannabis legalization (RCL), legalizing cannabis for non-medical, or recreational, purposes (also known as adult-use) (Marijuana n.d.). Most RCL states have regulated retail outlets, or dispensaries, where cannabis can be purchased legally, but they impose different controls such as taxation, licensing, and product testing (Wang and Wilson 2022, Tax Policy Center 2024, Hall et al. 2019). However, not all RCL states have operational recreational dispensaries (Minnesota, Virginia, and D.C., as of August 2025), creating a gap where consumption is legal, but purchase and sale remain illicit. Moreover, even though in 2023 over half of Americans lived in a state where the recreational use of cannabis was legal (Shah and Shah 2024), the majority of cannabis purchases in the United States continues to occur through unregulated, illicit sources (Kacey 2023, Cannabis Public Policy Consulting 2023). Cannabis obtained from non-regulated sources poses greater public health risks for various reasons including unknown and inconsistent potency, and higher risk of microbial and pesticide contamination (California 2022, Nieves 2021, Botel et al. 2021, Soboroff et al. 2022). Thus, decreasing the illicit market and shifting buyers to the regulated market represents a significant goal for public health (Gettman and Kennedy 2014, Cohen and McGowan 2012). 

### Prices in legal vs. illicit markets

The illicit market’s biggest advantage is price. Legal cannabis is generally more expensive than illicit cannabis (Wadsworth et al. 2022, Wadsworth et al. 2023, Goldstein et al. 2020, Munksgaard 2024), since illicit markets can avoid regulatory costs and taxation (Nieves 2021, Munksgaard 2024, Husock 2024). For example, in California, legal flower cost 11% more than unlicensed flower in 2019 (Goldstein et al. 2020). However, as the legal market within a state ages, prices of legal cannabis tend to decrease (Wadsworth et al. 2022, Smart et al. 2017, Caulkins et al. 2018, 2019 Recreational Marijuana Supply and Demand Legislative Report 2019, Carrel and Forbes 2023, Hammond and Goodman n.d.). In fact, consumer prices in Oregon fell from over $10 per gram in 2016 to less than $5 per gram in 2018 (2019 Recreational Marijuana Supply and Demand Legislative Report 2019). Such lower prices may encourage transition from the illicit market to the legal market (Hammond and Goodman n.d.), as price is an important factor for consumer choice (Nieves 2021, Hammond and Goodman n.d., Donnan et al. 2022, Shi et al. 2019, Goodman et al. 2022, Childs and Poirier 2021). Indeed, multiple behavioral economic studies in the U.S. and Canada have demonstrated that high legal pricing significantly increases the prevalence of illicit cannabis use. They consistently find that when legal cannabis is similarly priced to illicit options, consumers prefer the legal route, indicated by higher quantity demanded (2–4.5 g more) (Amlung and MacKillop 2019, Amlung et al. 2019). Although consumers may turn to illicit cannabis when legal prices rise, the legal market exerts a significantly stronger influence on the illicit market than vice versa. Specifically, the availability of the legal alternative makes illicit cannabis demand much more vulnerable to price changes – over nine times more so than the reverse effect (Amlung et al. 2019). Together, this suggests that efficient legalization can create a powerful policy lever: by strategically setting legal cannabis prices and taxes to remain competitive with illicit markets, regulators can shift consumer demand toward the legal market, thereby reducing reliance on illicit cannabis.

Ultimately, the successful undermining of the illicit market depends directly on the accessibility and cost of cannabis from legal dispensaries. Based on a discrete choice experiment with over 900 adults who use cannabis in RCL states, policy simulations predicts that as legal prices drop, the illicit market share will also fall (Xing and Shi 2024). However, survey data from the International Cannabis Policy Study showed that illicit market prices significantly increased in RCL states without dispensaries (Wadsworth et al. 2023), highlighting the importance of not just legalization, but also timely market access through regulated outlets.

### Study aims

Price benchmarks of cannabis are essential to inform policy and regulatory adjustments that ensure competitive legal market pricing and current literature presents three interrelated gaps. First, although Goldstein et al. compared legal and illicit prices within California (Goldstein et al. 2020), no study has yet performed an analysis that directly compares legal and illicit cannabis prices contemporaneously across multiple U.S states. Second, the most recent examination of how legalization influences illicit market prices used data from 2019, leaving a gap during which many states have legalized cannabis (Meinhofer and Rubli 2021, Davis et al. 2016, Malivert and Hall 2013, Caulkins and Bond 2012). Third, existing comparative studies rely on self-reported data for legal purchases (Wadsworth et al. 2023, Munksgaard 2024), introducing potential social desirability bias: respondents may overreport legal sourcing and underreport illicit sourcing due to the stigmatization and criminalization of cannabis use (Johnson 2014). Consequently, the true price differential between legal and illicit cannabis remains empirically unknown at a multi-state level with more recent data.

Thus, our aim in this study was to (1) compare illicit market cannabis flower prices using crowdsourced data from the Price of Weed (http://www.priceofweed.com*)* from 2018 to 2024 between all 50 states and D.C., and (2) compare these illicit cannabis flower prices with prices of cannabis flower from legal sources, using data from state agencies, within multiple RCL states (Colorado, Oregon, California, Massachusetts, Michigan, Illinois, Connecticut) to determine how dispensary pricing compares to pricing in the illicit market. By combining a comprehensive 50 state illicit price analysis with state agency comparisons of legal versus illicit prices in seven RCL states, this study fills the post-2019 empirical gap in illicit prices and explores whether and where legal markets have become price-competitive with illicit sources. Understanding the price differential is important for policymakers designing effective strategies to minimize the illicit market and maximize the potential benefits of legalization.

## Methods

### Illicit cannabis price data source: PriceofWeed.com

Our primary source for illicit cannabis data was Price of Weed (POW) (http://www.priceofweed.com*)*, a crowdsourced website that allowed users to anonymously submit cannabis prices by self-described quality, amount (ranging from 3.5 to 28 g), and location (city, state, country) with the intention of providing a global price index for the “street value of cannabis.” It displayed the mean price per country or state along with the five most recent submissions. Several previous studies have used this website to estimate illicit market prices (Meinhofer and Rubli 2021, Davis et al. 2016, Malivert and Hall 2013, Caulkins and Bond 2012). Increasing confidence in the representativeness of these prices, research on self-reported data of cannabis transactions suggests that the price of the most recent purchase and the average prices of the past three purchases do not differ (Bond et al. 2014). 

We developed two separate scraping pipelines in R to extract: (1) price submissions from the current version of the POW site, and (2) historical data from archived snapshots of the same site spanning 2018 to 2024 accessed via the Internet Archive Wayback Machine (https://web.archive.org/*).* The submission date was assumed to be the purchase date. Observations from Puerto Rico were excluded due to insufficient submissions (i.e. submissions per 100,000 population < 1).

#### Scraper

To collect the submissions from all 50 U.S. states and the District of Columbia, we created a web scraper to access the live version of the U.S. POW page, and hyperlinks to each state’s individual price report were extracted using HyperText Markup Language (HTML) parsing techniques. A custom function then visited each state-specific page to extract the most recent submissions (See *Supplemental Appendix A*). We first piloted this in February 2025, extracting the 219 most recent submissions from the POW website, with purchase dates ranging from 11/15/2019 to 12/27/2024. We then obtained historical pricing data by combining the same state-level URLs with the Internet Archive’s Capture Index Application Programming Interface (CDX API) to retrieve all available snapshots between January 1, 2018 and December 31, 2024 from the Wayback Machine. The scraper extracted 28,587 submissions from the Wayback Machine, with purchase dates ranging from 1/1/2018 to 12/27/2024. Submissions were determined to be duplicates and were removed if they had the same purchase date, price, quantity, and location, and different snapshot dates. We preserved duplicate submissions within individual snapshots because it cannot be distinguished whether they were submitted by the same or different users.

#### Final dataset

Price per gram was calculated for each submission and used for the analyses to improve comparability between submissions. To minimize measurement error from possible incorrectly entered submissions, we excluded prices per gram greater than or equal to $100 (*n* = 128) due to implausibility, in line with previous research using POW submissions (Davis et al. 2016). Otherwise, outliers were not removed.

In the final analytic sample, there were 15,235 submissions from all 50 states and D.C. with purchase dates ranging from 1/1/2018 to 12/27/2024. Overall, submissions per year steadily decreased from 5,588 submissions in 2018 to 3,469 submissions in 2020. Afterwards, there was a steep decline to approximately 369 submissions per year from 2021 to 2024. This decrease could be due to reduced POW submissions and/or reduced Internet Archive snapshots. However, when adjusting for mean state population during the study period, submission rates remained robust: the three states with the lowest submissions per 100,000 people (Texas, California, Florida) were among the top five states in terms of the highest absolute number of submissions (See Supplemental Tables 1 and 2).

### Legal cannabis price data

To estimate the prices of recreational cannabis flower purchased legally, we used public data from state government websites. The following data were publicly available: daily mean price per gram from Massachusetts (Cannabis Control Commission Massachusetts 2024); monthly mean price per gram from Colorado (Colorado Marijuana Enforcement Division n.d.), California (California Department of Cannabis Control n.d.), Michigan (Michigan Cannabis Regulatory Agency n.d.), and Connecticut (State of Connecticut n.d.); yearly mean price per gram from Maine (Maine Office of Cannabis Policy n.d.); monthly median price per gram from Oregon (Oregon Liquor and Cannabis Commission n.d.). We contacted RCL states without price data publicly available and received further data from Illinois to allow calculations of daily mean price per gram. Unfortunately, other states did not provide adequate data to estimate price per gram. Thus, the final analysis of illicit and legal prices included only the aforementioned states (Colorado, Oregon, California, Massachusetts, Michigan, Illinois, Maine, and Connecticut). Notably, legal estimates also differ by state regarding inclusion of taxes and discounts (See *Supplemental Table 3*).

### Analysis

#### Comparing illicit prices by recreational status in all 50 states and D.C.

First, we compared illicit cannabis prices by state recreational dispensary status. The primary outcome for this analysis was medians due to right skewness (Table 1). We determined recreational dispensary status by the presence or absence of recreational dispensaries for the majority of a given year in a state. This classification provides a more accurate depiction of the availability of legal cannabis in a state than using the RCL date. Thus, if a state’s first recreational cannabis dispensary opened before July 1 in a given year, the state was classified as “recreational” for that given year and those subsequent. Conversely, if a state’s first recreational cannabis dispensary opened on or after July 1, the state was not classified as “recreational” until the following year. Every year, median illicit cannabis prices were not significantly different between states with medical dispensaries and states with no dispensaries (See *Supplemental Table 4*), so they were combined into a “non-recreational” category. Wilcoxon rank sum tests were utilized to determine the significance of difference by recreational dispensary status, as prices were non-normally distributed.

**Table 1:** Illegal cannabis flower price per gram (USD) in all 50 States and D.C. by recreational dispensary status

	Recreational^1^	2018	2019	2020	2021	2022	2023	2024	Overall
Median (Q1, Q3)	Yes	$7.14 (4.29, 10.00)	$7.14 (5.00, 10.71)	$7.86 (5.36, 10.71)	$7.14 (5.36, 12.32)	$7.14 (3.84, 10.00)	$7.14 (4.29, 10.00)	$5 (3.57, 8.75)	$7.14 (4.64, 10.71)
	No	$10 (7.14, 11.43)	$8.93 (6.43, 11.43)	$9 (7.14, 11.43)	$8.93 (7.14, 10.71)	$8.57 (6.12, 11.43)	$8 (5.36, 10.71)	$7.14 (5.00, 8.93)	$8.93 (7.14, 11.43)
*P*-value		> 0.001	> 0.001	> 0.001	> 0.001	> 0.001	> 0.001	> 0.001	> 0.001

^1^ Recreational dispensary status determined for each state by presence or absence of recreational dispensaries for the majority of a given year

#### Comparing legal vs. illicit prices within eight recreational states (Colorado, Oregon, California, Massachusetts, Michigan, Illinois, Maine, and Connecticut)

Next, we compared illicit prices to legal dispensary prices. Despite illicit prices being skewed, means were used to compare prices between sources because most legal data was only available as means. Thus, for all states except Oregon, mean prices per gram per year were calculated from monthly (or daily) mean prices per gram to compare prices over the study period. Since Oregon could only provide monthly median prices per gram, medians were calculated for POW data from Oregon. For overall legal price statistics, daily mean prices per gram are averaged to create monthly means before creating yearly means to give equal weight to each state, while Oregon is excluded. We utilized Wilcoxon rank sum tests to determine the significance of difference by source.

## Results

### Illicit prices

**Fig. 1:**
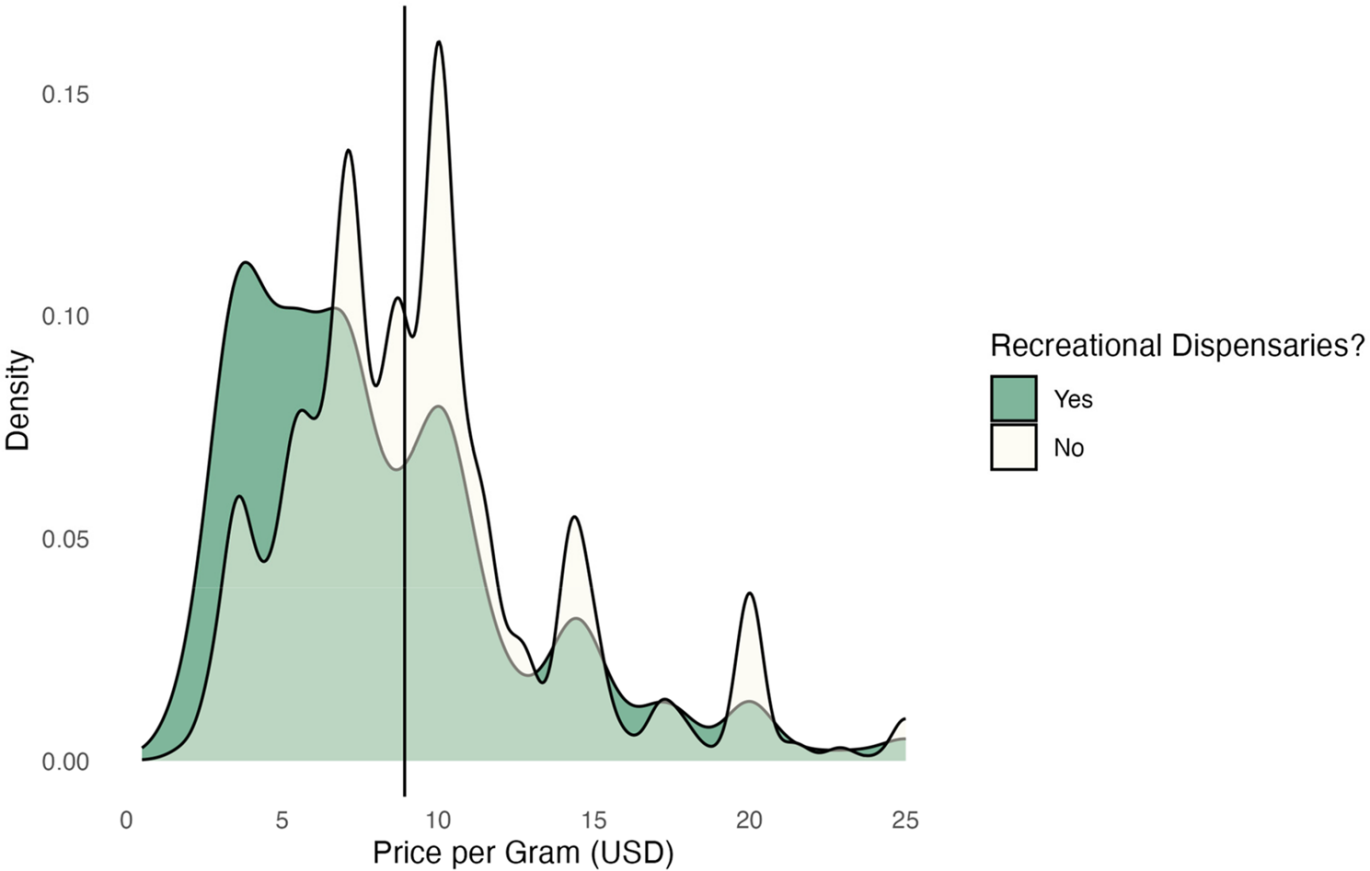
Density of illicit cannabis prices in all 50 states and D.C. compared to the U.S. median (2018–2024). Caption: *The range is bounded at $25/gram in this density chart. Prices above this correspond to 2.87% of prices in states without recreational dispensaries and 3.35% of prices in states with dispensaries*,* respectively. The majority of illicit cannabis prices per gram in states with recreational dispensaries were below the overall median price per gram ($8.93)*,* denoted by the solid vertical line. In contrast*,* purchases in states without recreational dispensaries tend to be above the median*

The overall median price per gram of illicit cannabis was significantly lower in recreational states ($7.10), compared to non-recreational states over the entire study period ($8.90, *p* < 0.001; Fig. 1). This price difference was consistent within each year as well, with the median prices decreasing from 2018 to 2024 (Table 1). The largest price decrease was between 2023 and 2024 for illicit cannabis from recreational states. Moreover, among RCL states, states with recreational dispensaries had a significantly lower median price per gram than states without ($7.10 vs. $10.00, *p* < 0.001) over the entire study period.

**Fig. 2:**
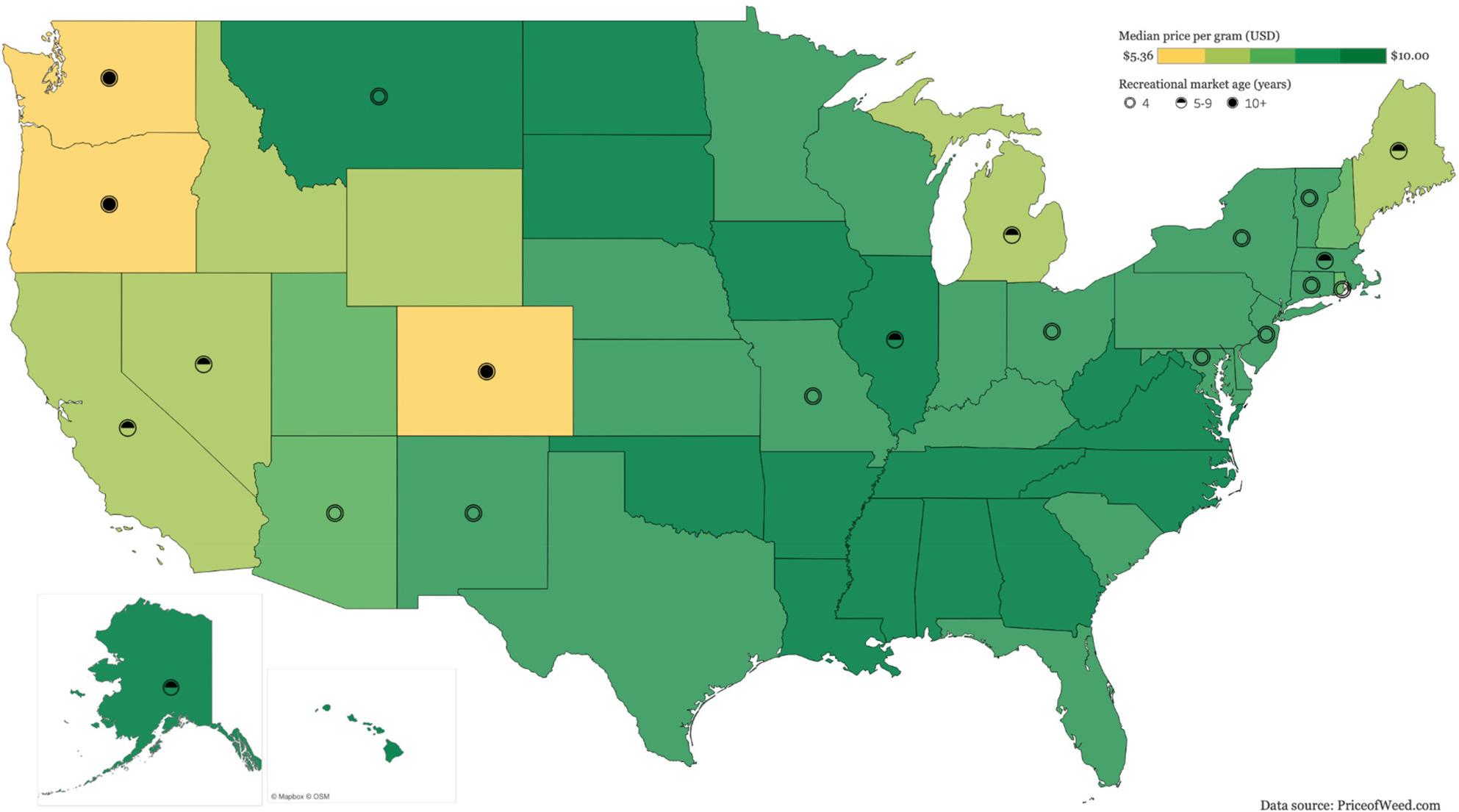
Median price per gram of illicit cannabis across 2018-2024. Caption: This map compares the median price per gram of illicit cannabis flower over 2018 to 2024 by state. States with recreational dispensaries are marked by symbols denoting market age: a solid circle indicates markets in 2014-2015; a half-filled circle indicates those opened in 2016-2020; and an unfilled circle indicates those opened in 2021-2024

The median price per gram of illicit cannabis over 2018–2024 is depicted by state in Fig. 2. All five states with the lowest median prices currently have recreational dispensaries. Further, the three states with the lowest overall median illicit prices – Oregon ($5.36), Washington ($5.71), Colorado ($6.07) – have the oldest legal recreational markets and had dispensaries throughout the entire study period. In contrast, among states with median prices per gram greater than or equal to $10, 80% lack recreational dispensaries. Figure 2 also suggests clustering effects, with non-recreational states near recreational states tending to have lower prices.

### Illicit vs. legal prices within recreational states

The overall mean price per gram of illicit and legal cannabis among seven RCL states (Colorado, California, Massachusetts, Michigan, Illinois, Maine, and Connecticut) when recreational dispensaries were operational in each state is presented in Table 2. From 2018 to 2024, states joined our sample as they emerged with recreational markets and available data. Despite the changing sample, mean illicit prices declined over the study period. On the other hand, legal prices fluctuated as new recreational markets were created. Overall, mean illicit prices were higher than legal prices ($9.53 vs. $8.43), although not significantly. In three out of the four years that showed significant differences (2020, 2021, 2022), illicit prices were significantly lower than legal prices.

**Table 2:** Mean price per gram (USD) of cannabis flower in states with recreational dispensaries

Year^1^	Illicit^2^,^3^	Legal^2^	States^4^
2018*	8.88 (6.08)	6.1 (3.42)	CO, MA≠
2019	10.3 (6.97)	9.43 (5.17)	CO, MA
2020*	9.66 (6.97)	11.91 (4.82)	CO, CA, MA, IL, MI≠, ME≠
2021*	9.73 (6.30)	10.75 (3.75)	CO, CA, MA, IL, MI, ME
2022*	7.55 (8.27)	8.1 (3.54)	CO, CA, MA, IL, MI, ME
2023	8.72 (12.73)	6.88 (2.95)	CO, CA, MA, IL, MI, ME, CT
2024	8.5 (8.86)	6.51 (3.17)	CO, CA, MA, IL, MI, ME, CT
Overall	9.53 (7.20)	8.43 (4.17)	

^1^*, *p*<0.05. Based on *p*-values from Wilcoxon rank sum test

^2^ Mean (Standard deviation)

^3^ Prices only included in means if state had recreational dispensaries and legal price data available at time of submission

^4^≠, state not included for full year. [MA joins 11/1/2018; MI joins 10/1/2020; ME joins11/1/2020]

**Fig. 3:**
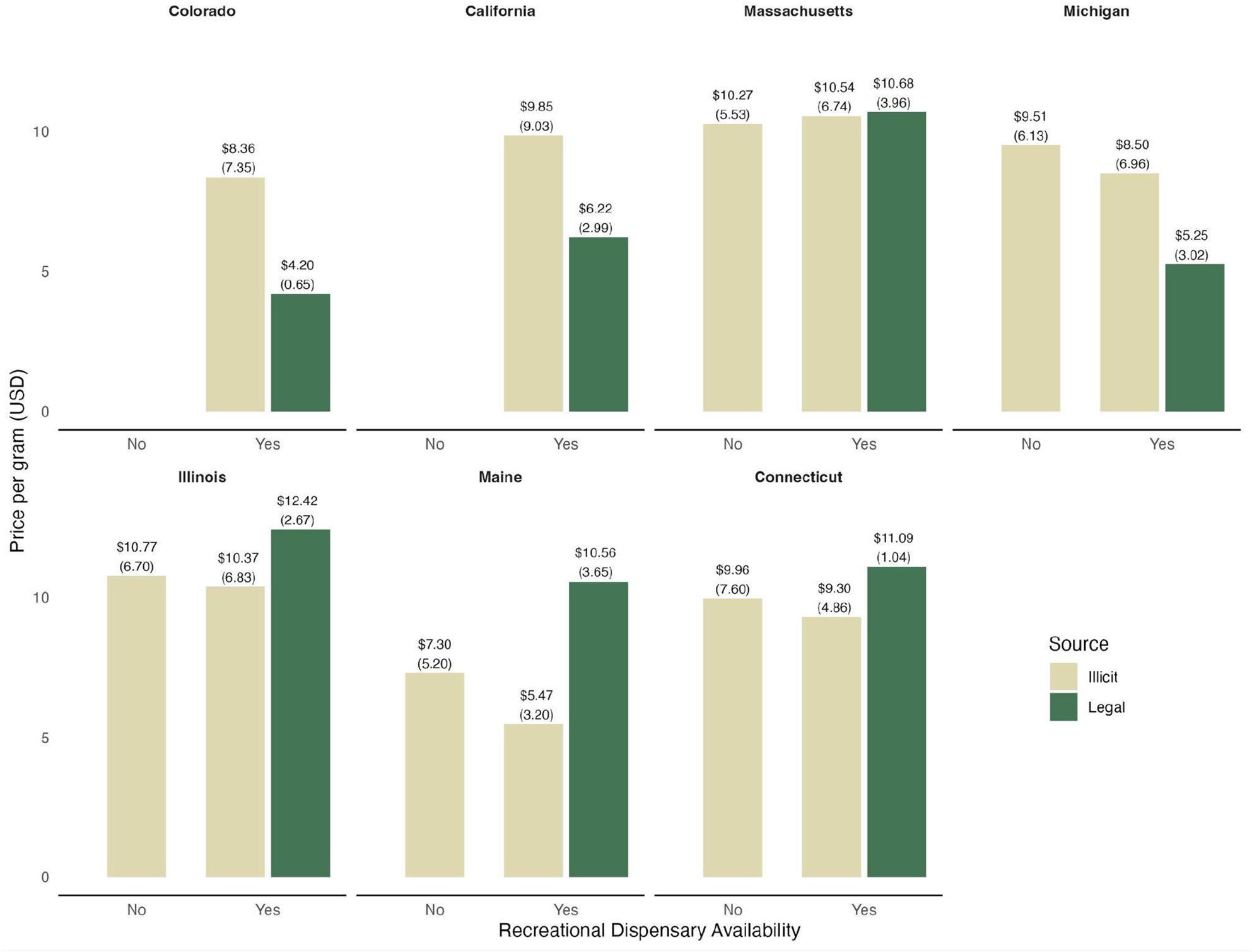
Mean price per gram (USD) of illicit and legal cannabis flower in states with recreational dispensaries (2018-2024). Mean price (Standard deviation). Caption: Barplots by state comparing cannabis prices across different legal time periods and sources. Data on illicit cannabis prices are drawn from *PriceofWeed.com*. Data on legal prices are from state agencies

Illicit and legal cannabis prices varied between states as well. The four states where overall, legal prices exceeded illicit prices (Massachusetts, Illinois, Maine, Connecticut) were among the five newest recreational markets (Fig. 3, *Supplemental* Table 3). Illinois had both the highest illicit and legal mean price per gram among the states sampled. In contrast, the three states with the lowest illicit cannabis prices also had legal prices significantly below illicit prices.

## Discussion

By incorporating multi-year, multi-state price data and comparing prices within states before and after the introduction of recreational dispensaries, this study adds to the growing evidence on the association between legally regulated cannabis and illicit cannabis prices in the U.S. Overall, we find that illicit market prices are lower in states that have recreational dispensaries and that the price ranking of legal versus illicit cannabis within a given state differs depending on the maturity of that state’s legal market. For example, while Colorado’s recreational market opened in 2014 and had legal prices approximately $3 less than illicit prices, Maine’s market opened in 2020 and had legal prices greater than $3 more than illicit prices.

Across 2018–2024, the median price of illicit cannabis from recreational states was 20.2% lower than that of illicit cannabis from states without recreational dispensaries. This difference is consistent with previous research (Wadsworth et al. 2023, Meinhofer and Rubli 2021), and further supports legalization and market implementation as a strategy to undermine the illicit market (Gettman and Kennedy 2014, Cohen and McGowan 2012). Further, the case of Washington D.C. suggests that legalization alone may have limited effects on illicit markets: although enacting RCL in 2015, Congress has consistently prevented the establishment of a regulated cannabis market (Flynn 2022), and D.C. had the highest illicit median price in our study. Without alternatives from accessible, legal outlets, the demand for illicit cannabis likely remains unchanged, or may even increase when consumption is decriminalized. This underscores the need for legal recreational dispensaries, and that simple legalization of consumption is not enough for public health benefits. Our findings reinforce this point: RCL states with recreational dispensaries had prices that were 40.8% lower than RCL states without recreational dispensaries.

Overall, in our sample of recreational states, there was not a significant difference between legal cannabis and illicit cannabis prices. However, in three out of the four years that showed significant differences, illicit prices were significantly lower than legal prices. When comparing prices at the state level, this legal price advantage was only seen in half of the sample, all except Michigan having the oldest legal markets. Although these findings are descriptive, below we consider components of the changing recreational landscape that may help situate these findings in the broader policy context. Since states that implemented RCL earlier tend to license cannabis businesses at lower cost and in greater volumes (Wang and Wilson 2022), increased competition and supply may be responsible for driving legal prices down, even below illicit prices. The lack of licensing caps in Michigan further supports this theory (Michigan Regulation and Taxation of Marihuana Act 2018). Additionally, market maturity could be contributing to the price advantage seen among Colorado, Oregon, and California, as self-report data from the International Cannabis Policy Study suggests that the odds of purchasing legally increased by 48% each year after legal dispensaries opened, among U.S. RCL states with dispensaries in 2019 (Wadsworth et al. 2023). 

In contrast, Massachusetts and Illinois, whose markets opened in November 2018 and January 2020, respectively, saw legal prices remain significantly higher than illicit prices for most years. Massachusetts experienced a delayed rollout, opening its recreational market almost two years after enacting RCL (Pardo 2020), and Illinois has strict market entry policies and high taxation (Hernandez 2024, Newell 2022). These findings imply that initial regulatory burdens, limited market entrants, and high taxation may hinder the ability of legal markets to compete on price, potentially slowing the transition of consumers away from the illicit market. In fact, in a differences-in-differences study with POW data from 2010 to 2019, Meinhofer and Rubli found that states with higher rates of licensed dispensaries and lower licensing fees experienced the greatest declines in illicit cannabis prices (Meinhofer and Rubli 2021). However, we found that recent declines in legal prices by over 50% in Massachusetts from 2022 to 2024 were paralleled by decreases in illicit prices (*Supplemental Table 3*), suggesting that post-implementation changes in legal market pricing can directly influence the illicit market. Continued research should explore the specific mechanisms that facilitate these price differentials.

Other factors that may influence today’s recreational pricing are the duration and maturity of a state’s prior medical market. States with longer-established markets, such as California, New Mexico, and Colorado which established medical cannabis outlets in the 2000s, tend to have more developed cultivation infrastructure and supply chains (Wang and Wilson 2022), which may lower recreational prices relative to states with shorter or more restrictive medical histories. Consequently, the medical market’s historical footprint represents an important contextual variable when comparing legal versus illicit price differentials across states and further research may wish to look at this.

Importantly, legal cannabis priced significantly higher than illicit cannabis perpetuates inequitable access to safer substances, thereby increasing the risks associated with consumption in price-sensitive, and often, more vulnerable populations, such as young adults (Hammond and Goodman n.d.). Closing these price gaps helps incentivize a shift towards safer, regulated products and in contrast, may reduce risks, such as criminal activity and exposure to contaminated products. It would also be financially beneficial for states, as other studies have shown that people who consume cannabis daily are less likely to purchase legally (Wadsworth et al. 2022, Hammond and Goodman n.d.). Attracting these high-spending customers to dispensaries could support the long-term viability of legal markets by rerouting revenue from illicit competitors to states, schools, and local governments via tax revenue (Tax Policy Center 2024, Ingraham 2016). In fact, it’s estimated that each year, on average over one hundred million dollars of tax revenue are lost per illicit state (Cannabis Public Policy Consulting 2023). Thus, competitively priced legal cannabis may promote public health. However, it is important to consider that lower cannabis prices could have mixed public health implications. Although reduced prices may encourage consumers to transition from illegal to regulated markets, lower prices may also increase cannabis use and initiation, as has been observed for alcohol and tobacco products (Xu and Chaloupka 2011, Chaloupka et al. 2011). Future work should evaluate both the potential benefits and unintended consequences of increasingly affordable cannabis markets.

### Strengths and Limitations

A strength of this study is the use of state agency-reported data, increasing the precision of legal price estimates. The diversity among the included states in terms of timing and regulatory contexts allowed for a nuanced comparison of illicit and legal cannabis prices. Moreover, the anonymity of users submitting illicit cannabis prices on POW likely reduced social desirability bias, possibly increasing accuracy. However, this advantage also presents some challenges, as crowdsourced data can be subject to recall or sampling bias. Individuals who choose to submit prices may be systematically different from people who consume cannabis and do not submit data. If so, this illicit price data would not be generalizable to the U.S. population. Additionally, sample sizes for illicit purchases varied between states and decreased over time, which may reduce the precision of state-level annual estimates and limit our ability to detect differences between illicit and legal purchases in later study years. Nonetheless, when we aggregated the data, consistent trends emerged that align with previous research. Furthermore, although cannabis flower is the most comparable item between legal and illicit sources and is used by 74% of people who use cannabis (Substance Abuse and Mental Health Services Administration 2024), differences in quality or THC potency, or the lack of non-flower products (including intoxicating hemp products) in our estimations could still influence price comparisons. For flower in both the illicit and legal markets, quantity discounts exist (Munksgaard 2024), but we could not account for them in our analysis due to data constraints. Likewise, other state-level factors, such as taxation and discounts, may also influence legal pricing patterns without being fully captured in our analysis.

For all sources, prices were not standardized to 2024 inflated prices. However, this decision likely did not impact our findings since prices decreased across markets. Finally, lack of standardized price data from legal states limited our sample. We recommend increased surveillance and transparency of state-level cannabis data to aid evaluation of these new markets.

## Conclusion

Our findings suggest that legal cannabis prices in more mature recreational markets are often closer to illicit cannabis prices than those observed in newer recreational markets, where legal cannabis frequently remains substantially more expensive than illicit cannabis. These patterns may have important implications for the ability of regulated markets to compete with illicit sources. Policymakers should consider how taxation, regulation, and market structure may influence the relative affordability of legal cannabis as recreational markets continue to evolve. Future research should examine how changes in legal cannabis pricing relate to consumer purchasing behaviors, illegal market participation, and broader public health outcomes.

## Supplementary Information

Supplementary Material 1

Supplementary Material 2

## Data Availability

The datasets used and/or analyzed during the current study are available from the corresponding author on reasonable request.

## Abbreviations

RCL: Recreational Cannabis Legalization
POW: PriceofWeed.com
THC: Delta-9-tetrahydrocannabinol

## References

[CR21] 2019 Recreational Marijuana Supply. and Demand Legislative Report. Portland, Oregon: Oregon Liquor Control Commission; 2019 [cited 2025 Sep 10]. Available from: https://www.oregon.gov/olcc/marijuana/Documents/Bulletins/2019%20Supply%20and%20Demand%20Legislative%20Report%20FINAL%20for%20Publication(PDFA).pdf

[CR28] Amlung M, MacKillop J. Availability of legalized cannabis reduces demand for illegal cannabis among Canadian cannabis users: evidence from a behavioural economic substitution paradigm. Can J Public Health. 2019;110(2):216–21. 10.17269/s41997-018-0160-4. 30523535 10.17269/s41997-018-0160-4 PMC6964395

[CR29] Amlung M, Reed DD, Morris V, Aston ER, Metrik J, MacKillop J. Price elasticity of illegal versus legal cannabis: a behavioral economic substitutability analysis. Addiction. 2019;114(1):112–8. 10.1111/add.14437. 30194789 10.1111/add.14437 PMC13235258

[CR36] Bond B, Caulkins JP, Scott N, Kilmer B, Dietze P. Are users’ most recent drug purchases representative? Drug Alcohol Depend. 2014;142:133–8. 10.1016/j.drugalcdep.2014.06.016. 25008105 10.1016/j.drugalcdep.2014.06.016

[CR10] Botel D, Boudreau A, Rackov A, Rehman A, Phillips B, Hay C et al. Analysis of Illicit and Legal Cannabis Products for a Suite of Chemical and Microbial Contaminants. New Brunswick Research and Productivity Council; 2021 [cited 2026 Jan 4]. Available from: https://businessofcannabis.com/wp-content/uploads/2021/10/Comparison-of-Illicit-and-Legal-Cannabis-Samples-1.pdf

[CR39] California Department of Cannabis Control. Sales and Price per Unit Report. [cited 2026 May 12]. Available from: https://www.cannabis.ca.gov/resources/data-dashboard/daily-sales-units-by-item-category-report/

[CR8] California I. the world’s largest legal weed market is going up in smoke. The Economist. 2022 [cited 2025 Oct 2]. Available from: https://www.economist.com/united-states/2022/05/14/in-california-the-worlds-largest-legal-weed-market-is-going-up-in-smoke

[CR37] Cannabis Control Commission Massachusetts. Daily Average Price Per Gram for Adult-Use Cannabis. 2024 [cited 2026 May 12]. Available from: https://masscannabiscontrol.com/open-data/data-catalog/

[CR7] Cannabis Public Policy Consulting. Percent of Total Cannabis That is Regulated in the United States. Cannabis Public Policy Consulting; 2023 [cited 2025 Nov 4]. Available from: https://www.cannabispublicpolicyconsulting.com/wp-content/uploads/2023/09/2023-June-RDCOS-Factsheet.pdf

[CR22] Carrel L, Forbes. 2023 [cited 2025 Aug 25]. Cannabis Market Has Matured To Point Of Declining Sales Growth. Available from: https://www.forbes.com/sites/lcarrel/2023/06/12/cannabis-market-has-matured-to-point-of-declining-sales-growth/

[CR20] Caulkins JP, Bao Y, Davenport S, Fahli I, Guo Y, Kinnard K, et al. Big data on a big new market: insights from Washington State’s legal cannabis market. Int J Drug Policy. 2018;57:86–94. 10.1016/j.drugpo.2018.03.031. 29709847 10.1016/j.drugpo.2018.03.031 PMC6948109

[CR34] Caulkins JP, Bond BM. Marijuana Price Gradients: Implications for Exports and Export-Generated Tax Revenue for California After Legalization. J Drug Issues. 2012;42(1):28–45. 10.1177/0022042612436650.

[CR51] Chaloupka FJ, Straif K, Leon ME. Effectiveness of tax and price policies in tobacco control. Tob Control. 2011;20(3):235–8. 10.1136/tc.2010.039982. 21115556 10.1136/tc.2010.039982

[CR27] Childs J, Poirier A. Implications of marijuana purchase task based demand functions for optimal legal pricing of cannabis. Int J Drug Policy. 2021;95:103271. 10.1016/j.drugpo.2021.103271. 34049233 10.1016/j.drugpo.2021.103271

[CR13] Cohen E, McGowan R. Grass is Always Greener When It’s Legal: Policies for State Regulated Marijuana. Econ Voice. 2012;9(1). 10.1515/1553-3832.1923.

[CR38] Colorado Marijuana Enforcement Division. Colorado MED Dashboard. [cited 2026 May 12]. Available from: https://public.tableau.com/app/profile/cu.business.research.division/viz/ColoradoMEDDashboard/Overview

[CR32] Davis AJ, Geisler KR, Nichols MW. The price elasticity of marijuana demand: evidence from crowd-sourced transaction data. Empir Econ. 2016;50(4):1171–92. 10.1007/s00181-015-0992-1.

[CR24] Donnan J, Shogan O, Bishop L, Swab M, Najafizada M. Characteristics that influence purchase choice for cannabis products: a systematic review. J Cannabis Res. 2022;4(1):9. 10.1186/s42238-022-00117-0. 35105374 10.1186/s42238-022-00117-0 PMC8805380

[CR44] Flynn M. The Washington Post (Online). Washington, D.C., United States: WP Company LLC d/b/a The Washington Post; 2022 [cited 2025 Nov 21]. Congress keeps ban on legal D.C. marijuana sales in budget, despite Democratic control. Available from: https://www.proquest.com/docview/2637478525/citation/1336F99D562D4E96PQ/1

[CR12] Gettman J, Kennedy M. Let it grow—the open market solution to marijuana control. Harm Reduct J. 2014;11(1):32. 10.1186/1477-7517-11-32. 25403234 10.1186/1477-7517-11-32 PMC4247667

[CR16] Goldstein RS, Saposhnik R, Sumner DA. Prices of Cannabis in California from Licensed and Unlicensed Retailers. ARE Update. 2020;23(3). Available from: https://s.giannini.ucop.edu/uploads/giannini_public/70/d1/70d16c7a-3e45-4769-8e6c-f78e2c6f3135/v23n3_1.pdf

[CR26] Goodman S, Wadsworth E, Hammond D. Reasons for Purchasing Cannabis From Illegal Sources in Legal Markets: Findings Among Cannabis Consumers in Canada and U.S. States, 2019–2020. J Stud Alcohol Drugs. 2022;83(3):392–401. 10.15288/jsad.2022.83.392. 35590180

[CR4] Hall W, Stjepanović D, Caulkins J, Lynskey M, Leung J, Campbell G, et al. Public health implications of legalising the production and sale of cannabis for medicinal and recreational use. Lancet. 2019;394(10208):1580–90. 10.1016/S0140-6736(19)31789-1. 31657733 10.1016/S0140-6736(19)31789-1

[CR23] Hammond D, Goodman S. Analysis of drivers of the illicit cannabis market.

[CR47] Hernandez A. Big Business Runs Illinois Cannabis. South Side Weekly. 2024 Apr 12 [cited 2025 Oct 13]. Available from: https://southsideweekly.com/big-business-runs-illinois-cannabis/

[CR18] Husock H. How Should State and Local Governments Respond to Illegal Retail Cannabis?. American Enterprise Institute; 2024 [cited 2025 Nov 4]. Available from: https://www.jstor.org/stable/resrep57612?seq=1

[CR49] Ingraham C, Study. States are losing out on billions of dollars by keeping pot illegal. Washington Post – Blogs. 2016 [cited 2025 Oct 2]. Available from: https://www.proquest.com/docview/1789129693/citation/CA4B708D2DC34FBDPQ/1

[CR35] Johnson TP. Sources of error in substance use prevalence surveys. Int Sch Res Not. 2014;2014:923290. 10.1155/2014/923290. 10.1155/2014/923290 PMC4897110 27437511

[CR6] Kacey M, Washington DC. New Frontier Data; 2023 [cited 2025 Aug 29]. Available from: https://3324860.fs1.hubspotusercontent-na1.net/hubfs/3324860/Reports/NFD-2023USCannabisReport.pdf?utm_campaign=2023%20US%20Cannabis%20Report&utm_medium=email&_hsenc=p2ANqtz-9UGfTruDFuvf8O6tQklEf9T49pJRpCMYx9Y9FZhuuZtXYYA2Bbb2GmG-7B5AWC4ejAATLwPEeItDcCBB_ZEzW6lEJXZQ&_hsmi=247984940&utm_content=247984940&utm_source=hs_automation

[CR42] Maine Office of Cannabis Policy. Adult Use Retail Sales Data. [cited 2026 May 12]. Available from: https://app.powerbigov.us/view?r=eyJrIjoiOTAyZGUxNWYtMWNhZS00OTAzLTlhNTUtMjBhNTU3NjMwN2QzIiwidCI6IjQxM2ZhOGFiLTIwN2QtNGI2Mi05YmNkLWVhMWE4ZjJmODY0ZSJ9

[CR33] Malivert R, Hall JC. The effect of medical marijuana laws on extralegal marijuana prices. Atl Econ J. 2013;41(4):455–6. 10.1007/s11293-013-9384-0.

[CR1] Marijuana Policy Project. MPP. [cited 2025 Nov 4]. Cannabis Legalization. Available from: https://www.mpp.org/issues/legalization/

[CR31] Meinhofer A, Rubli A. Illegal drug market responses to state recreational cannabis laws. Addiction. 2021;116(12):3433–43. 10.1111/add.15517. 33998087 10.1111/add.15517 PMC8578142

[CR40] Michigan Cannabis Regulatory Agency. Marijuana Regulatory Agency Statistical Report. [cited 2026 May 12]. Available from: https://www.michigan.gov/cra/resources/cannabis-regulatory-agency-licensing-reports/cannabis-regulatory-agency-statistical-report

[CR45] Michigan Regulation and Taxation of Marihuana Act. Section 333.27959. 2018 Dec 6. Available from: https://www.legislature.mi.gov/Laws/MCL?objectName=mcl-333-27959

[CR17] Munksgaard R. Between Main Street and Illicit Markets: Self- Reported Prices and Source Premiums in US Cannabis Markets. In. Montreal, Quebec, Canada; 2024.

[CR48] Newell N. The Illinois cannabis social equity program: Where it went wrong and how it can be fixed. Chic-Kent Law Rev. 2022;97(2):601–24.

[CR9] Nieves A. California’s legal weed industry can’t compete with illicit market. POLITICO. 2021 [cited 2025 Nov 4]. Available from: https://www.politico.com/news/2021/10/23/california-legal-illicit-weed-market-516868

[CR43] Oregon Liquor and Cannabis Commission. Harvest, Price, & Sales Market Data. [cited 2026 May 12]. Available from: https://www.oregon.gov/olcc/marijuana/pages/marijuana-market-data.aspx

[CR46] Pardo B. In: Decorte T, Lenton S, Wilkins C, editors. Legalizing cannabis: experiences, lessons and scenarios. 1 ed. Abingdon New York (NY): Routledge; 2020. p. 17.

[CR5] Shah AC. and S. Most Americans now live in a legal marijuana state – and most have at least one dispensary in their county. Pew Research Center. 2024 [cited 2025 Aug 29]. Available from: https://www.pewresearch.org/short-reads/2024/02/29/most-americans-now-live-in-a-legal-marijuana-state-and-most-have-at-least-one-dispensary-in-their-county/

[CR25] Shi Y, Cao Y, Shang C, Pacula RL. The impacts of potency, warning messages, and price on preferences for Cannabis flower products. Int J Drug Policy. 2019;74:1–10. 10.1016/j.drugpo.2019.07.037. 31382201 10.1016/j.drugpo.2019.07.037 PMC6893125

[CR19] Smart R, Caulkins JP, Kilmer B, Davenport S, Midgette G. Variation in cannabis potency & prices in a newly-legal market: evidence from 30 million cannabis sales in Washington State. Addiction Abingdon Engl. 2017;112(12):2167–77. 10.1111/add.13886. 10.1111/add.13886 PMC5673542 28556310

[CR11] Soboroff J, Koss M, Lee J, Lozano AV, Heikkila A. Raids on black market cannabis farms uncover human trafficking victims. NBC News. 2022 [cited 2026 May 7]. Available from: https://www.nbcnews.com/news/us-news/raids-black-market-cannabis-farms-uncover-human-trafficking-victims-rcna46787

[CR41] State of Connecticut. Cannabis Average Price Per Gram. [cited 2026 May 12]. Available from: https://data.ct.gov/Health-and-Human-Services/Cannabis-Average-Price-Per-Gram/ttwq-xhyz

[CR52] Substance Abuse and Mental Health Services Administration, Center for Behavioral Health Statistics and Quality. 2024 National Survey on Drug Use and Health. 2024. Available from: https://www.samhsa.gov/data/data-we-collect/nsduh-national-survey-drug-use-and-health/datafiles

[CR3] Tax Policy Center. 2024 [cited 2025 Sep 25]. How do state and local cannabis (marijuana) taxes work? Available from: https://taxpolicycenter.org/briefing-book/how-do-state-and-local-cannabis-marijuana-taxes-work

[CR14] Wadsworth E, Driezen P, Pacula RL, Hammond D. Cannabis flower prices and transitions to legal sources after legalization in Canada, 2019–2020. Drug Alcohol Depend. 2022;231:109262. 10.1016/j.drugalcdep.2021.109262. 34998249 10.1016/j.drugalcdep.2021.109262

[CR15] Wadsworth E, Driezen P, Pacula RL, Kilmer B, Hammond D. Prices and purchase sources for dried cannabis flower in the United States, 2019–2020. Cannabis Cannabinoid Res. 2023;8(5):923–32. 10.1089/can.2021.0232. 35363550 10.1089/can.2021.0232

[CR2] Wang LX, Wilson NJ. U.S. state approaches to cannabis licensing. Int J Drug Policy. 2022;106:103755. 10.1016/j.drugpo.2022.103755. 35691088 10.1016/j.drugpo.2022.103755

[CR30] Xing J, Shi Y. Cannabis consumers’ preferences for legal and illegal cannabis: evidence from a discrete choice experiment. BMC Public Health. 2024;24(1):2397. 10.1186/s12889-024-19640-1. 39227852 10.1186/s12889-024-19640-1 PMC11373389

[CR50] Xu X, Chaloupka FJ. The effects of prices on alcohol use and its consequences. Alcohol Res Health. 2011;34(2):236–45. 22330223 PMC3860576

