# Supplementary material for: High stakes: how legal cannabis pricing impacts the illicit market
Source: J Cannabis Res. 2026 Jun 6;8:93. doi: 10.1186/s42238-026-00455-3 (PMC13471419; doi:10.1186/s42238-026-00455-3)
Supplement: Supplementary file 1 [file 42238_2026_455_MOESM1_ESM.docx]

Appendix A

Real-time scraper

- All state pages have similarly structured URLs starting with <http://www.priceofweed.com/prices/United-States/> and then followed by “State.”
  - Main USA URL, <https://www.priceofweed.com/prices/United-States.html>, was saved as an object called ‘main_url.’
  - The HTML content of the main page was read using the read_html function from the ‘rvest’ package and then saved as ‘main_page.’
  - To pull data from all state pages, the state URLs were extracted from ‘main_page.’
    - First by extracting all <a> tags using html_nodes
    - Then getting the href attribute using html_attr
    - Filtering for state-specific URLs and excluding city-specific URLs
    - ‘<https://www.priceofweed.com>’ was added to then create the vector called ‘state_urls’
- Several custom functions …
  - Function read the last scraped date from a file
    - This was to try to get it to run repeatedly and only add things if new submissions had been posted. It did work, but it isn’t necessary and isn’t even necessarily helpful given the low speed with which new submissions are posted to the website currently.
    - Thinking that it will be overkill to explain
  - Function to save the last scraped date to a file
    - Similar to above
  - Function to scrape the latest submission from the data table
    - This custom function reads
    - The HTML content of each state page was read from each state_url using the read_html function from the ‘rvest’ package.
      - , which is used for static web scraping
    - The bottom table was targeted using the html_element function from the ‘rvest’ package and selecting “table.table”, as determined by inspecting the elements from the webpage.
  - Function to clean and format the scraped data
- The custom function scrape_latest_submissions was used on state_urls and the data was bound together using map_dfr from the ‘purr’ package.

Archive scraper

- Custom functions
  - Scrape url for latest
    - Using GET from the httr package,
    - HTML content parsed using read_html (rvest package)
    - Initialize table variable as NULL
    - Two types of website structure
      - Before 03/21/2021
        - Prior to early 2021, the latest submissions table had the 15 latest submissions.
        - It was targeted using html_node/html_element (rvest), selecting "//h3[contains(text(), 'Submissions')]/following-sibling::table[1]”
      - New structure after early 2021.
        - Only 5 rows of latest submissions shared on page
        - Also different element structure – table with class = “table”
  - Fetch all snapshots
    - This function retrieved all archived snapshots of a specific URL from the Internet Archive’s Wayback Machine using the CDX API.
      - The CDX API is a query system for the Internet Archive
        - “The wayback-cdx-server is a standalone HTTP servlet that serves the index that the wayback machine uses to lookup captures.” [Wayback CDX Server API - BETAInternet Archivehttps://archive.org › developers › wayback-cdx-server](https://archive.org/developers/wayback-cdx-server.html)
      - Important since the states do not have the same snapshot dates.
    - The CDX API URL was constructed by adding the Wayback archive url stem to state_url
      - Asks for all available snapshots of the given state_url, and requests the output in JSON format.
    - Use jsonlite::fromJSON() to send the request and parse the response (jsonlite)
      - Result is a list of archived snapshots
    - Timestamps extracted from this list and returned as a vector
    - To handle missing data or errors: If no snapshots found, it logs a warning and returns NULL
    - tryCatch() block catches any errors and returns NULL with a warning message.
    - Summary: Given a URL for a state page on [PriceofWeed.com](http://priceofweed.com), this function asks the Internet Archive: “When have you saved this page?” It then collects and returns a list of those timestamps so that later parts of the script can download and analyze the page content from each of those archived dates.
  - Filter snapshots by date range
    - This function filters all the timestamps that were pulled by the previous custom function by a given date range, saving them as a vector called filtered_timestamps.
- Set up
  - To run this code, first the start and end dates were stated as January 1, 2018 to March 8, 2025
  - A list was initialized to store successful archives using list() from base R.
  - A new environment was created as a hash table for processed hashes (called processed_hashes)
  - State urls creation
    - A states vector was created with all the states
      - Format ex: “New-Hampshire”
    - URLs for states were generated first by setting the base_url to be <http://www.priceofweed.com/prices/United-States/> and then combining the base_url with the states vector to create state_urls
- Scraping loops
  - Overall goal: loop through state’s [PriceOfWeed.com](http://priceofweed.com) page, grab all archived versions from the Wayback Machine within a certain date range, scrape the price submission tables, and save the results into a single CSV file.
  - A loop was set up to iterate through each state page (each state_url in state_urls)
  - Pause before fetching snapshot for 2 seconds to avoid overloading the server
  - Custom function ‘fetch_all_snapshots’ was used on each state_url to get a list of timestamps when this page was archived, saving the results as the vector, all_snapshots
  - Only continue if the Wayback Machine actually has snapshots for this state page.
  - These snapshots were then filtered by ‘filter_snapshots_by_date’ to filter snapshots between the stated start_date and end_date.
  - Another loop was initiated to iterate through the filtered snapshots
    - For each timestamp in filtered_snapshots, closest_state_url was created by pasting the Internet Archive stem (<https://web.archive.org/web/>), timestamp, “/”, and the state_url
    - This builds the full archived URL to scrape.
  - Then ‘scrape_wayback_url_4latest’ tries to read and parse the table of price submissions from the snapshot, saving it as scraped_table.
  - Checking that the table was scraped successfully
    - If the scrape exists, but has zero rows, skip it
    - All scraped tables were added to table_hash using digest from the digest package. “sha256” was selected as the hash function algorithm to compute a compact digest of the serialized object.
      - This creates a unique hash based on the table content and URL and is used to detect duplicate tables.
    - Check if table was already seen
      - If the table hasn’t been added before, it is marked as seen and added to the list of successful tables, which is called successful_subs_archives
    - Otherwise, skip duplicate
  - Code to print error messages if table is missing or no snapshots were found
  - Then 4 second pause before starting the next state to prevent overload
  - All collected tables in sucessful_subs_archives are merged into one dataframe called all_archive_subs, using bind_rows() (dplyr package)
  - Lastly, all_archive_subs was saved as a CSV
